# Supplementary material for: Genome-wide mapping of Vibrio cholerae VpsT binding identifies a mechanism for c-di-GMP homeostasis
Source: Nucleic Acids Res. 2021 Dec 15;50(1):149–59. doi: 10.1093/nar/gkab1194 (PMC8754643; doi:10.1093/nar/gkab1194)
Supplement: gkab1194_Supplemental_Files [file gkab1194_supplemental_files.zip › 03112021_paper_supplementary_figures.pptx]

## Slide 1
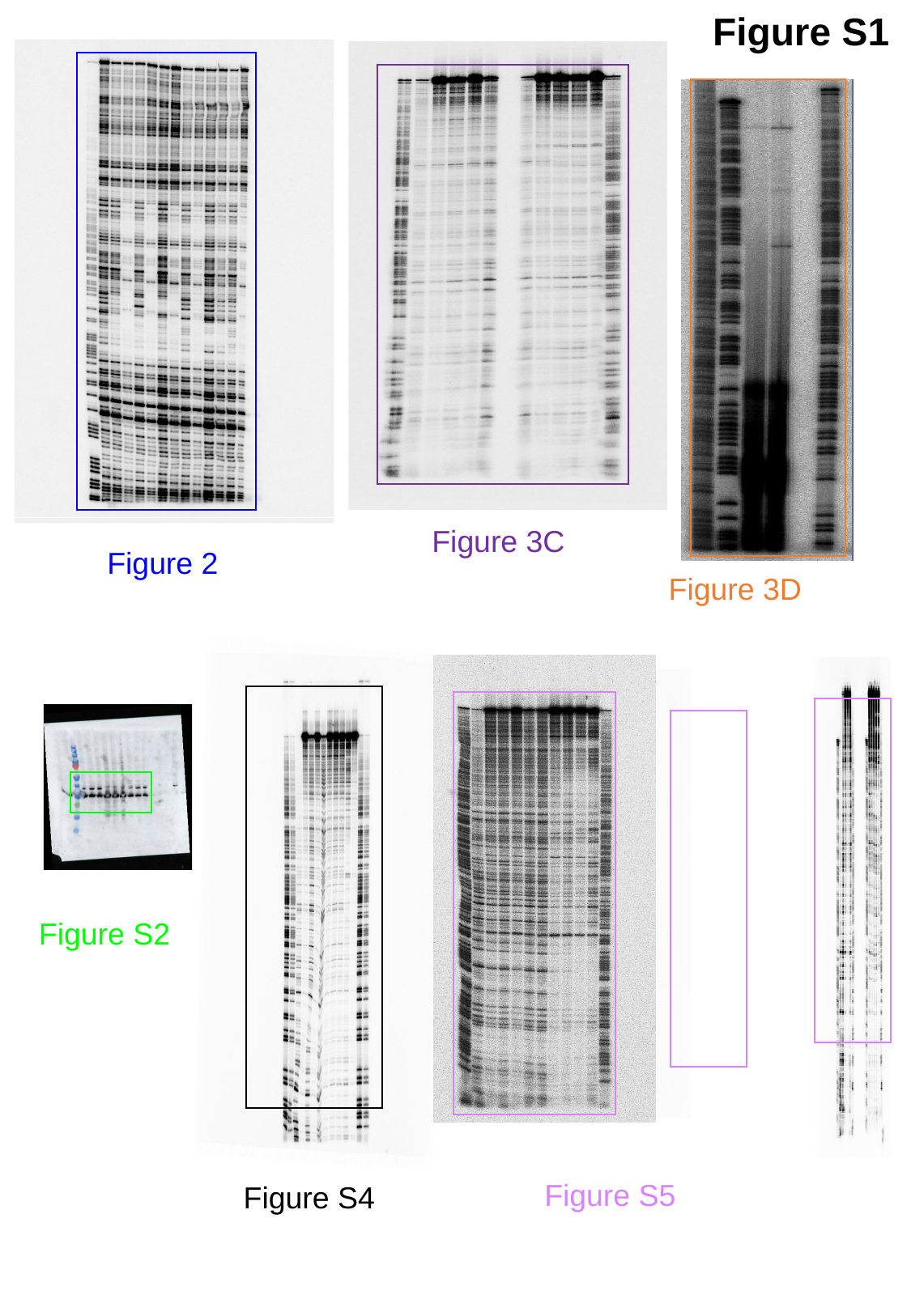

Figure S1
Figure 3C
Figure 2
Figure 3D
Figure S2
Figure S5
Figure S4

## Slide 2
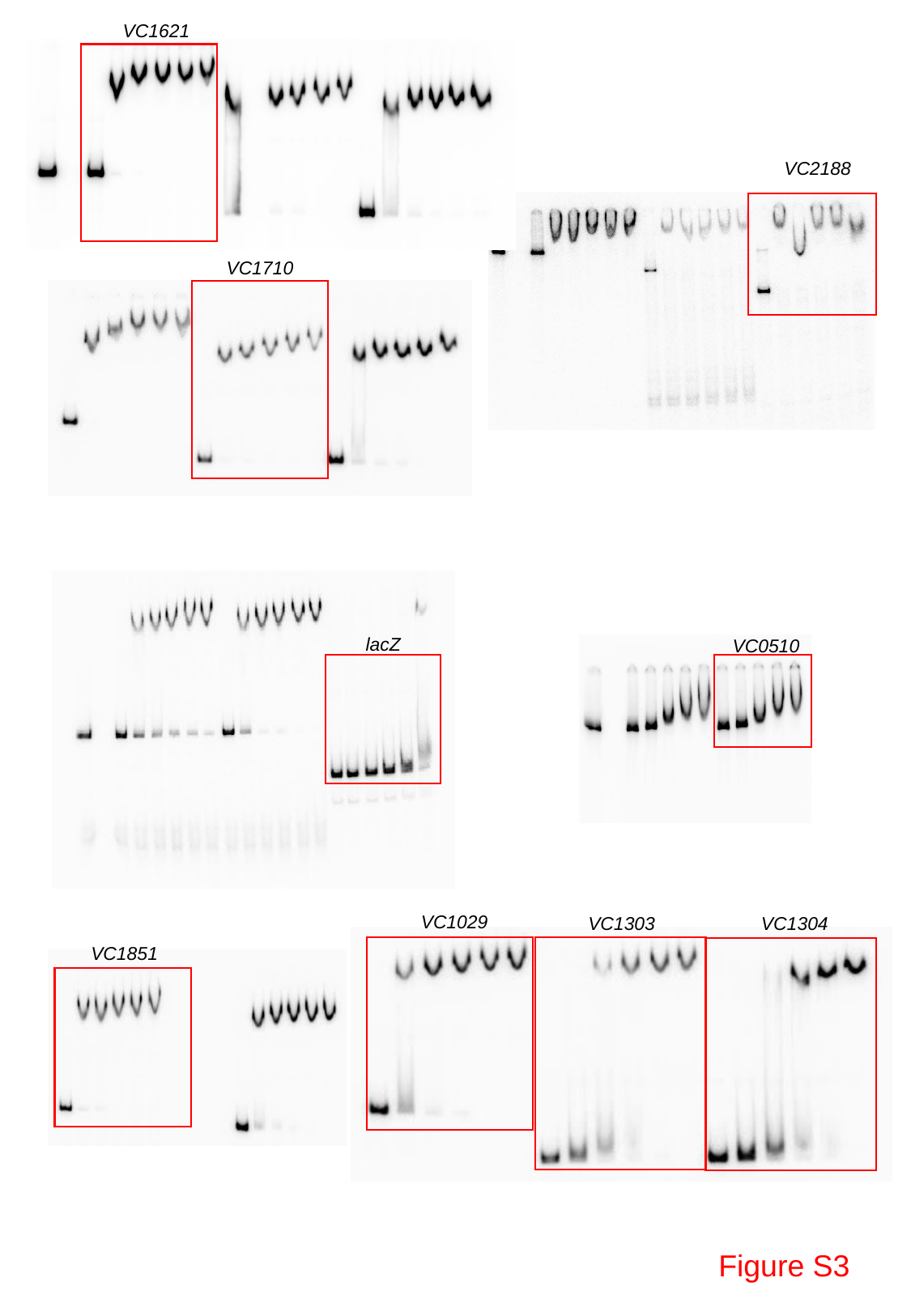

VC1621
VC2188
VC1710
lacZ
VC0510
VC1029
VC1304
VC1303
VC1851
Figure S3

## Slide 3
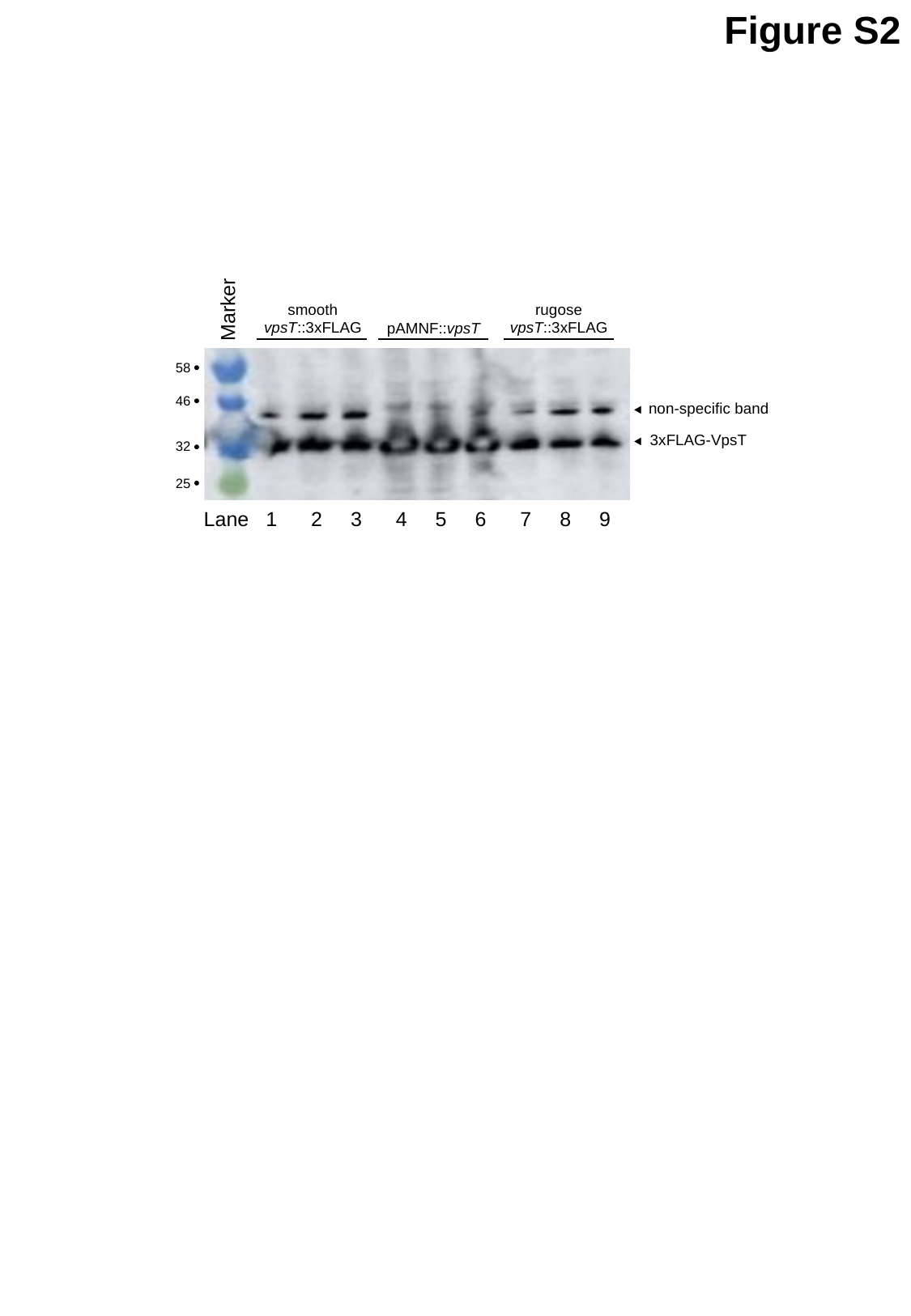

Figure S2
Marker
smooth
vpsT::3xFLAG
rugose
vpsT::3xFLAG
pAMNF::vpsT
58
46
non-specific band
‣
3xFLAG-VpsT
‣
32
25
Lane 1 2 3 4 5 6 7 8 9

## Slide 4
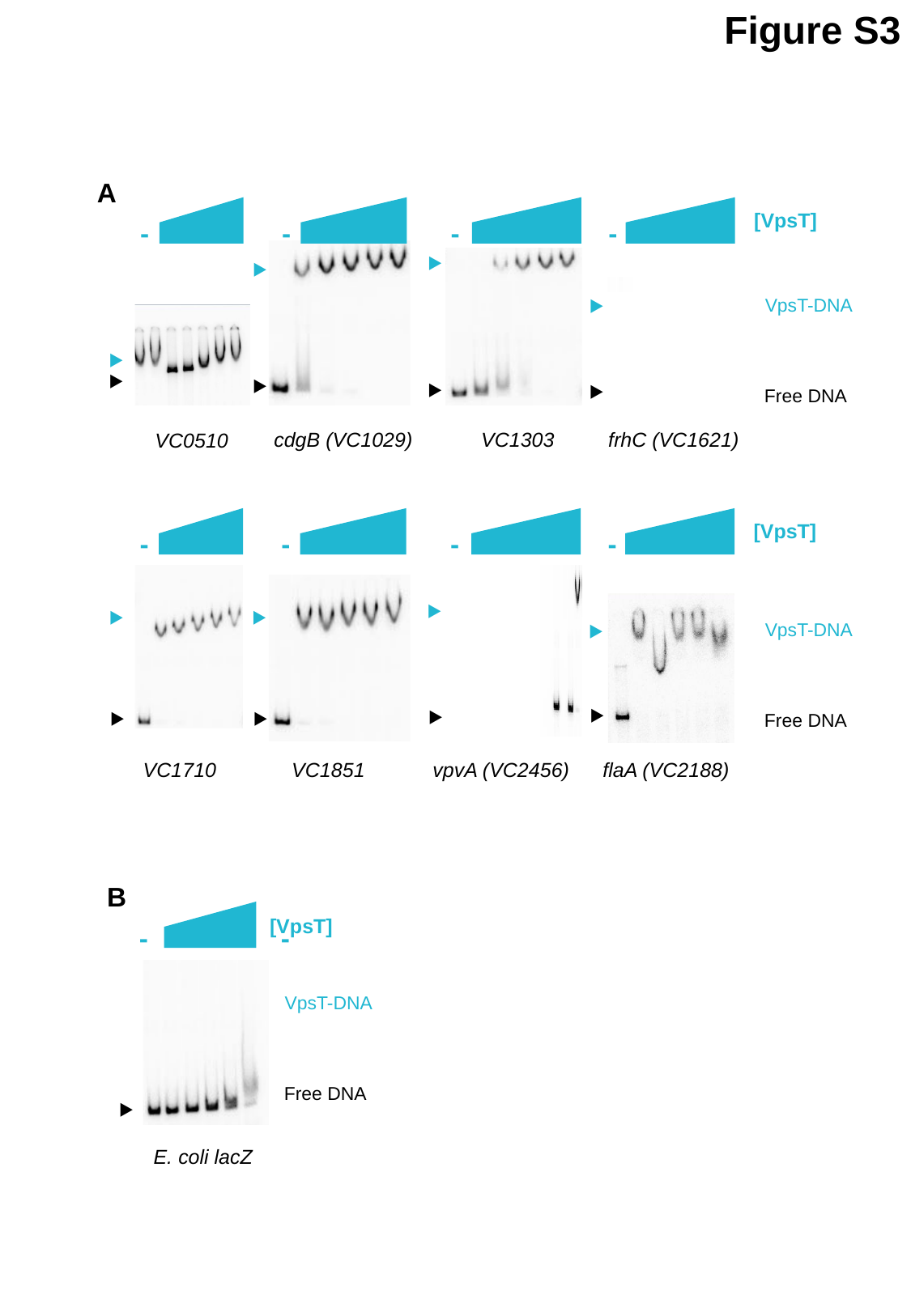

Figure S3
A
[VpsT]
-
-
-
-
‣
‣
‣
VpsT-DNA
‣
‣
‣
‣
‣
Free DNA
VC0510
cdgB (VC1029)
 VC1303
frhC (VC1621)
[VpsT]
-
-
-
-
‣
‣
‣
‣
VpsT-DNA
‣
‣
‣
‣
Free DNA
VC1710
VC1851
vpvA (VC2456)
flaA (VC2188)
B
[VpsT]
-
-
VpsT-DNA
Free DNA
‣
E. coli lacZ

## Slide 5
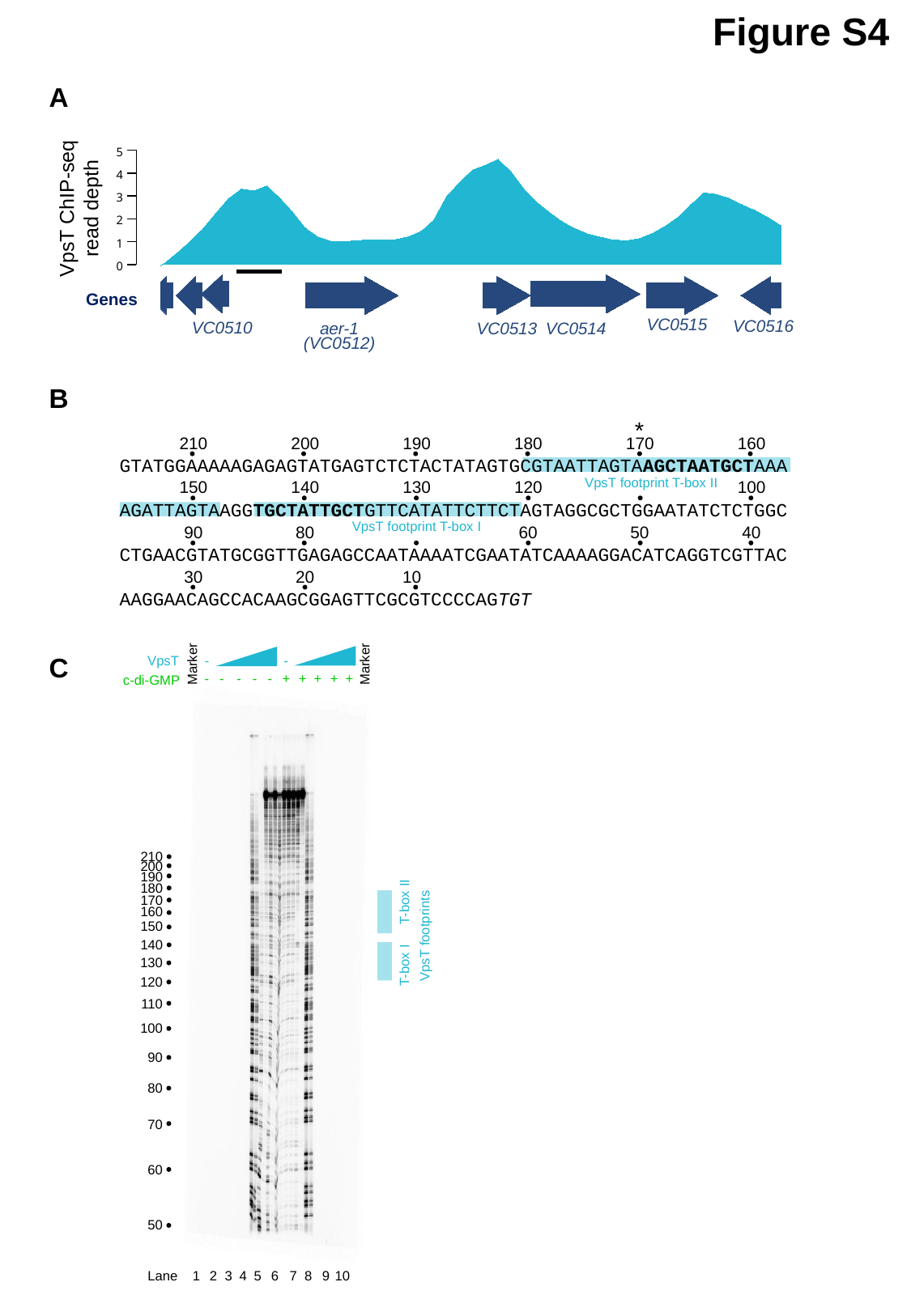

Figure S4
A
5
4
VpsT ChIP-seq read depth
3
2
1
0
Genes
VC0515
VC0516
VC0510
aer-1
(VC0512)
VC0513
VC0514
B
*
 210 200 190 180 170 160
 150 140 130 120 100
 90 80 60 50 40
 30 20 10
GTATGGAAAAAGAGAGTATGAGTCTCTACTATAGTGCGTAATTAGTAAGCTAATGCTAAA
AGATTAGTAAGGTGCTATTGCTGTTCATATTCTTCTAGTAGGCGCTGGAATATCTCTGGC
CTGAACGTATGCGGTTGAGAGCCAATAAAATCGAATATCAAAAGGACATCAGGTCGTTAC
AAGGAACAGCCACAAGCGGAGTTCGCGTCCCCAGTGT
VpsT footprint T-box II
VpsT footprint T-box I
VpsT
-
-
Marker
Marker
+
-
-
-
-
-
+
+
+
+
c-di-GMP
210
200
190
180
170
T-box II
160
150
VpsT footprints
140
130
T-box I
120
110
100
90
80
70
60
50
Lane
1
2
3
4
5
6
7
8
9
10
C

## Slide 6
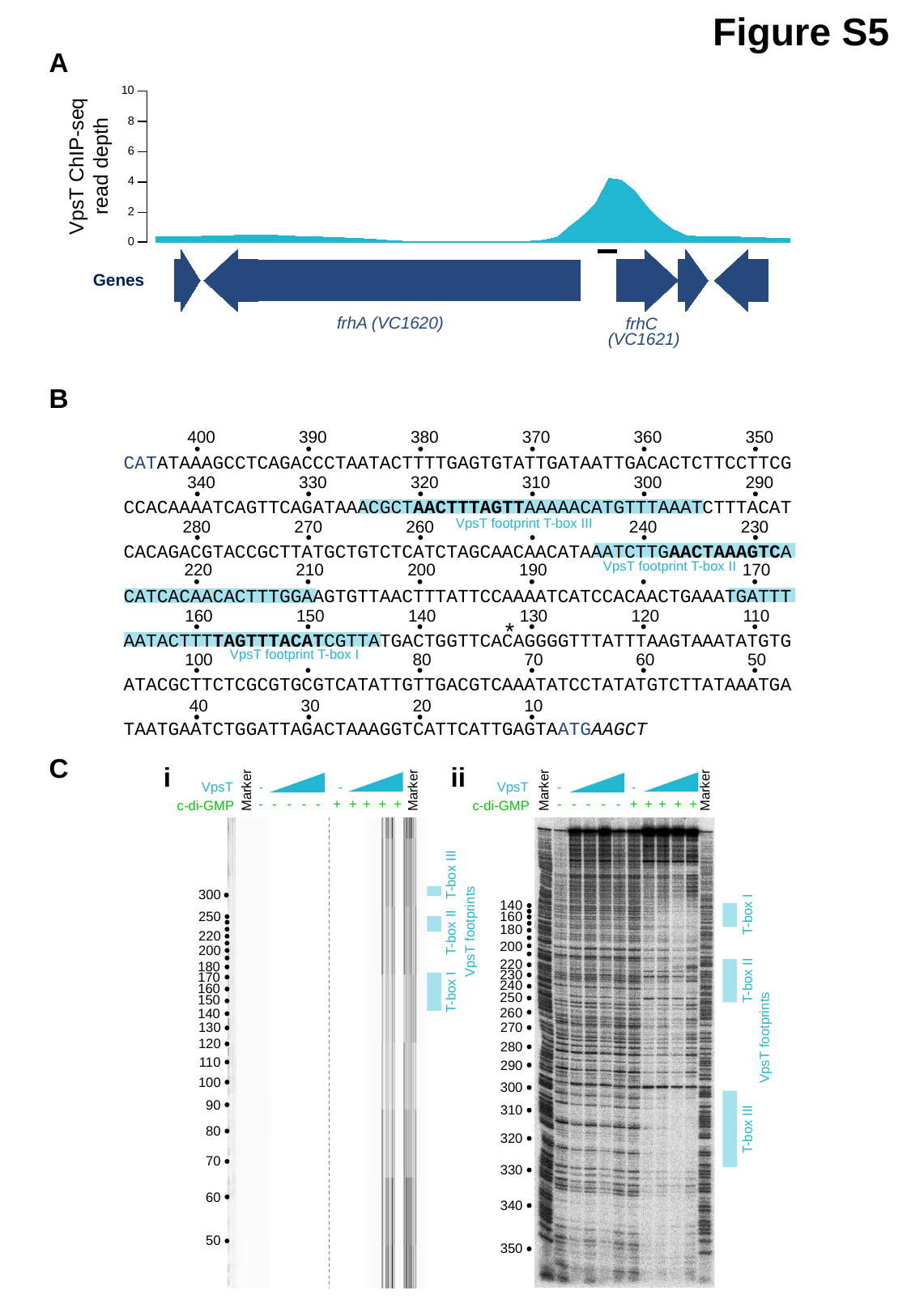

Figure S5
A
10
8
VpsT ChIP-seq read depth
6
4
2
0
Genes
frhA (VC1620)
frhC
(VC1621)
B
400 390 380 370 360 350
CATATAAAGCCTCAGACCCTAATACTTTTGAGTGTATTGATAATTGACACTCTTCCTTCG
CCACAAAATCAGTTCAGATAAACGCTAACTTTAGTTAAAAACATGTTTAAATCTTTACAT
CACAGACGTACCGCTTATGCTGTCTCATCTAGCAACAACATAAATCTTGAACTAAAGTCA
CATCACAACACTTTGGAAGTGTTAACTTTATTCCAAAATCATCCACAACTGAAATGATTT
AATACTTTTAGTTTACATCGTTATGACTGGTTCACAGGGGTTTATTTAAGTAAATATGTG
ATACGCTTCTCGCGTGCGTCATATTGTTGACGTCAAATATCCTATATGTCTTATAAATGA
TAATGAATCTGGATTAGACTAAAGGTCATTCATTGAGTAATGAAGCT
340 330 320 310 300 290
280 270 260 240 230
220 210 200 190 170
160 150 140 130 120 110
100 80 70 60 50
40 30 20 10
VpsT footprint T-box III
VpsT footprint T-box II
*
VpsT footprint T-box I
C
i
ii
VpsT
-
-
VpsT
-
-
Marker
Marker
Marker
Marker
+
+
-
-
-
-
-
+
+
+
+
-
-
-
-
-
+
+
+
+
c-di-GMP
c-di-GMP
T-box III
300
140
160
250
T-box I
180
220
VpsT footprints
T-box II
200
200
220
180
230
170
240
T-box II
160
250
T-box I
150
260
140
270
130
VpsT footprints
120
280
110
290
100
300
90
310
80
T-box III
320
70
330
60
340
50
350
