# Supplementary material for: Genome-wide mapping of Vibrio cholerae VpsT binding identifies a mechanism for c-di-GMP homeostasis
Source: Nucleic Acids Res. 2021 Dec 15;50(1):149–59. doi: 10.1093/nar/gkab1194 (PMC8754643; doi:10.1093/nar/gkab1194)
Supplement: gkab1194_Supplemental_Files [file gkab1194_supplemental_files.zip › Supplementary Figure legends.docx]

**SUPPLEMENTARY FIGURE LEGENDS**

**Figure S1:** Original gel images. Original uncropped gel images used for this work. Coloured boxes indicate the approximate boundaries of images following cropping of images for figures.

**Figure S2:** Comparison of VpsT levels in different strains. The figure shows the result of a western blotting experiment, with anti-FLAG antibodies, to detect chromosomal or plasmid encoded 3xFLAG-VpsT. Each measurement was done in triplicate for the strains indicated above the gel image.

**Figure S3:** Binding of purified VpsT *in vitro* to DNA targets identified by ChIP-seq *in vivo*. A) The images show results from electrophoretic mobility shift assays with purified VpsT (0-7.5 μM), c-di-GMP (50 μM) and DNA fragments corresponding to ChIP-seq peaks detected for VpsT binding *in vivo*. B) A negative control electrophoretic mobility shift assay demonstrating no binding of VpsT to *Escherichia coli* *lacZ* DNA.

**Figure S4:** VpsT binds two targets at the *aer* regulatory region. A) Binding of VpsT to the *aer* regulatory region *in vivo*. The VpsT ChIP-seq signal shown in cyan is the average of reads aligned from two independent experiments. Block arrows in navy blue indicate genes that are labelled by name and/or locus tag. The solid black bar indicates the location of the DNA sequence shown in panel B. B) DNA sequence of regulatory region upstream of *aer*. Bold typeface indicates the T-box consensus identified by ChIP-seq. The asterisk indicates the centre of the ChIP-seq peak for VpsT binding. The cyan box highlights the section of the regulatory region protected from DNAse I digestion by VpsT. C) Image of a denaturing polyacrylamide gel used to separate DNA fragments resulting from DNAse I digestion of the *aer* regulatory region. The gel is calibrated with a Maxam-Gilbert ‘G+A’ ladder. The presence and absence of VpsT (2, 4, 6 or 8 μM) and c-di-GMP (50 μM) is indicated.

**Figure S5:** VpsT binds three targets at the *frhA/frhC* regulatory region. A) Binding of VpsT to the *frhA*/*frhC* regulatory region *in vivo*. The VpsT ChIP-seq signal shown in cyan is the average of reads aligned from two independent experiments. Block arrows in navy blue indicate genes that are labelled by name and/or locus tag. The solid black bar indicates the location of the DNA sequence shown in panel B. B) DNA sequence of regulatory region upstream of *frhC*. Bold typeface indicates the T-box consensus identified by ChIP-seq. The asterisk indicates the centre of the ChIP-seq peak for VpsT binding. The cyan box highlights the section of the regulatory region protected from DNAse I digestion by VpsT. The *frhC* start codon is in blue text. c) Image of a two denaturing polyacrylamide gels used to separate DNA fragments resulting from DNAse I digestion of the DNA sequence upstream of *frhC*. The length of the regulatory DNA meant that it was necessary to label the 5' ends of either the (i) bottom or (ii) top DNA strands to get full coverage of the sequence. The gel is calibrated with a Maxam-Gilbert ‘G+A’ ladder. The presence and absence of VpsT (2, 4, 6 or 8 μM) and c-di-GMP (50 μM) is indicated. C) Image of a denaturing polyacrylamide gel used to separate DNA fragments resulting from DNAse I digestion of the *aer* regulatory region. The gel is calibrated with a Maxam-Gilbert ‘G+A’ ladder. The presence and absence of VpsT (2, 4, 6 or 8 μM) and c-di-GMP (50 μM) is indicated.
