# Supplementary material for: Genome-wide mapping of Vibrio cholerae VpsT binding identifies a mechanism for c-di-GMP homeostasis
Source: Nucleic Acids Res. 2021 Dec 15;50(1):149–59. doi: 10.1093/nar/gkab1194 (PMC8754643; doi:10.1093/nar/gkab1194)
Supplement: gkab1194_Supplemental_Files [file gkab1194_supplemental_files.zip › Table S1.docx]

**Table S1: Strains, plasmids and oligonucleotides**

**Name Description Source**

*Escherichia coli strains*

DH5⍺ *endA*1, *glnV*44, *thi*-1, *recA*1, *relA*1, *gyrA*96, *deoR*, *nupG*, *purB*20, NEB φ80d*lacZ*ΔM15, Δ(l*acZYA*-*argF*) U169, *hsdR*17(rK–mK+), λ

JCB387 Δ*nirB*, ∆*lac* (37)

S17 λpir *lac*U169 (*lacZ*M15), *recA*1, *endA*1, *hsdR*17, *thi*-1, *gyrA*96, *relA*1, *pir*

*Vibrio cholerae strains*

E7946 Wildtype *Vibrio cholerae* El Tor Ogawa derivative, SmR (33)

E7946 E7946 derivative lacking *vpsT* This work

Δ*vpsT*

E7946 E7946 derivative lacking *vpvABC* This work

Δ*vpvABC*

E7946 E7946 derivative with a 60 bp deletion that removes the *vpvA*P2 This work

Δ*vpvA*P2 promoter and most of the overlapping T-box I

E7946 E7946Δ*vpvA*P2 derivative with two point mutations in T-box II This work

Δ*vpvA*P2^Mut^

*Plasmids*

pET28a High copy number plasmid for expression of N-terminal His_6_-tagged Novagen proteins, KanR

pAMNF Plasmid for basal expression of N-terminal 3xFLAG-tagged proteins, (52) KanR

pRW50T A broad-host range *lacZ* expression vector encoding *oriT* of pRK, (38) TetR, Tra+

pBR322 General cloning vector, AmpR, TetR (53)

pSR 4 kb pBR322 derivative that encodes AmpR. Contains an *Eco*RI- (54) *Hin*dIII cloning site upstream of the λoop transcription terminator

pRK2013 Helper plasmid for conjugation, KanR, oriColE1, RK2-, Mob+, Tra+ (55)

pKAS32 Suicide plasmid for mutant strain construction, AmpR (35)

pFY4535 pFY4357 containing the *hok*/*sok* region from pXB300, GmR (46)

*Oligonucleotides for promoter cloning and mutagenesis (5' to 3')^1^*

VC0510|1.1F GGCTGCGAATTCCATTTACATTTGATTGGTATAATAATTCGC This work

VC0510|1.1R GCCCGAAGCTTCATTTCGGGCTCCTGTCGGAATCAATGG This work

VC0512|1.1R GCCCGAAGCTTCATTTCGGGCTCCTGTCGGAATCAATGG This work

VC0512|1.2F GGCTGCGAATTCCATTTCGGGCTCCTGTCGGAATCAATGG This work

VC1030|1.1F GGCTGCGAATTCTGACTTTCAGTGAGTCCATCCTGTTCTCC This work

VC1030|1.1R GCCCGAAGCTTCACCGACCTGATTCCTAATGAGTGATCGC This work

VC1031|1.1F GGCTGCGAATTCCACTGTCCGTACCTCCCCATGCTTCGATAT This work

ACACCCC

VC1031|1.1R GCCCGAAGCTTCATCTCACGCTGATTGAGTTAAGTGTTGT TC This work

AAAATGAGCC

VC1621|1.1F GGCTGCGAATTCCATATAAAGCCTCAGACCCTAATAC This work

VC1621|1.1R GCCCGAAGCTTCATTACTCAATGAATGACCTTTAGTC This work

VC1620|1.1R GCCCGAAGCTTCATATAAAGCCTCAGACCCTAATAC This work

VC1620|1.1F GGCTGCGAATTCCATTACTCAATGAATGACCTTTAGTC This work

VC1710|1.1R GCCCGAAGCTTCATCCGGTATCCCTAAACTGATGATG This work

VC1710|1.1F GGCTGCGAATTCCATATTTTGGCCTCAAGATTAAGAGGGC This work

VC1711|1.1F GGCTGCGAATTCCATCCGGTATCCCTAAACTGATGATG This work

VC1711|1.1R GCCCGAAGCTTCATATTTTGGCCTCAAGATTAAGAGGGC This work

VC1851|1.1R GCCCGAAGCTTCAAAGATTTACCTATTTTCGGGTTGTTG This work

VC1851|1.1F GGCTGCGAATTCACACCGCCCTGCAATTCACTCCATTC This work

VC2188|1.2F GGCTGCGAATTCCAGTTAGTTGAAGAATTTCATACCGCCTTT This work

ATCCATCTC

VC2188|1.1R GCCCGAAGCTTCATAGTTTGCTCTCCTATCGAGTTCGC This work

VC2456|1.1F GGCTGCGAATTCTGCGGCGTTCATGCTTTGGTTCATTTC This work

VC2456|1.1R GCCCGAAGCTTCAAGTGAAATATTCTCCATTTCGATCGTC This work

Δ*vpvA*P2|F GGCTGCGAATTCGTGCTTAATTTTCC This work

Δ*vpvA*P2^ΔT^|F GGCTGCGAATTCCTTTGCACCCAACG This work

Δ*vpvA*P2|R| GGGTGCAAAGAGTCATAAAATTCATTCTTCCGTTGG This work

G120|C115|

*vpsL*|wt|1.1|F GGCTGCGAATTCGTTCTGTTTTTCCTTTC This work

*vpsL*|wt|1.1|R GCCCGAAGCTTACTAGACGCTCCTAACC This work

*vpsL*|-213T|R TGAAAATAAACTTTAGTTTAATTTTATGATGGTTAATAGG This work

*vpsL*|-203T|R ATTAACCACCATGAAAATAAAATTTAGTTTACTTTTATGATGG This work

*vpsL*| -216C TAACCTATTAACCATCATACAAGTAAACTAAAGTTTATTT This work

*vpsL*| -208T TTAACCATCATAAAAGTAAATTAAAGTTTATTTTCATGGTG This work

*vpsL*| -200G ATAAAAGTAAACTAAAGTTGATTTTCATGGTGGTTAATAA This work

*Oligonucleotides for cloning N-terminal FLAG-tagged VpsT in pAMNF (5' to 3')*

VpsT|VC| GGCTGCGGTACCAAAGATGAAAACAAACTAAACGTTAGAAT This work

Nter|For GCTTTCTG

VpsT|VC| GCCCGAAGCTTTTAAGAATTGACTTCCTCAATTCCAATATT This work

Nter|Rev ATTTTTCGC

*Oligonucleotides cloning VpsT in pET28a (5' to 3')*

VpsT|Nhis|F CCCAATTCCATATGAAAGATGAAAACAAACTAAACGTTAGA This work

ATGCTTTC

VpsT|Nhis|R GGCGGATCCTTAAGAATTGACTTCCTCAATTCCAATATTAT This work

TTTTCGC

*Oligonucleotides to construct an N-terminal 3xFLAG chromosomal vpsT (5' to 3')*

up|flank|fwd TAGAGGTACCGGTTGTTAACTACTAAACGAAATTACCGTAT This work

C

up|flank|rev TGGAATAAATCATTTCACCCCTCCTAAC This work

down|flank| CCAAGGGTACCAAAGATGAAAACAAACTAAACG This work

fwd

down|flank| CGCCAGCTGCAGGCGGCCGCTAACATTTTTAAAGATCTGTT This work

rev TGC

flag|fwd GGGTGAAATGATTTATTCCAATGTCACACAC This work

flag|rev TTTCATCTTTGGTACCCTTGTCATCGTC This work

*Oligonucleotides used for MuGENT to delete vpsT (5' to 3')*

gw|Mugent| ACTCGAAGAGACATTACACAACGAATTCACCCCTCCTAACA This work

vpsT|F2| CATCAAGGC

RC+R1

gw|Mugent| GCCTTGATGTGTTAGGAGGGGTGAATTCGTTGTGTAATGTC This work

vpsT|R1| TCTTCGAGT

RC+F2

*Oligonucleotides for construction of mutations at the chromosomal vpvABC locus (5' to 3')*

pKAS32|fwd GCGGCCGCCTGCAGCTGG This work

pKAS32|rev GTTAACAACCGGTACCTCTAGAACTATAGCTAGCATGCGC This work

vpvA|p2| TTAGTGATTGGTGAGACAAAAATACCCGTTGGGTGCAAAG This work

deletion|m1| AGTCATAAAAT

exo|F1.1

pkAS32|vpv|f ATGGATGGTGATGCCATCCGGCGGCCGCCTGCAGCTGGCG This work

pkAS32|vpv|r AGTTGAGCAGTACATAGTCGCTCTGTACATGTCCGCGGTC This work

GCGACG

vpvA|arm1|f CTAGAGGTACCGGTTGTTAACGACTATGTACTGCTCAACT This work

TAGCG

vpvA|arm1|r ACGCCGTTAATCGCGAGAGGCTTCGAGC This work

vpvA|arm2|f CCTCTCGCGATTAACGGCGTTCAAGGATC This work

vpvA|arm2|r CGCCAGCTGCAGGCGGCCGCATGCAGCGGAAATTGCTG This work

vpvAp2| CTAGAGGTACCGGTTGTTAATTGAAGATTGATTTGCTCAA This work

arm1|f C

vpvAp2| ACGCCGTTAAGTGCTTAATTTTCCCCAAC This work

arm1|r

vpvAp2| AATTAAGCACTTAACGGCGTTCAAGGATC This work

arm2|f

vpvAp2| CGCCAGCTGCAGGCGGCCGCGGATGGCATCACCATCCATC This work

arm2|r

mvpvAp2| TAGAGGTACCGGTTGTTAACGCTAGAAAAAGCGCGCGC This work

arm1|r

^1^positions of point mutations are underlined
